# Current Advances in Nanoelectronics, Nanosensors, and Devices

**DOI:** 10.3390/nano14211771

**Published:** 2024-11-04

**Authors:** Filippo Giubileo

**Affiliations:** CNR-SPIN Salerno, via Giovanni Paolo II, n. 132, 84084 Fisciano, Italy; filippo.giubileo@spin.cnr.it

## Abstract

This Special Issue on “Current Advances in Nanoelectronics, Nanosensors, and Devices” collects cutting-edge research and comprehensive reviews in the rapidly evolving field of nanotechnology. This collection aims to highlight key breakthroughs in nanostructures, 2D materials, and their applications in nanoelectronics, nanosensors, and emerging device technologies.

This Special Issue on “Current Advances in Nanoelectronics, Nanosensors, and Devices”, collects cutting-edge research and comprehensive reviews in the rapidly evolving field of nanotechnology. This collection aims to highlight key breakthroughs in nanostructures, 2D materials, and their applications in nanoelectronics, nanosensors, and emerging device technologies. The development of nanoelectronics, nanosensors, and nanodevices is rapidly transforming our society, from industry to healthcare, and from energy to telecommunications and environmental monitoring [1].

Nanoelectronics enables the design and development of new devices with unprecedented performance, profiting from the unique properties of nanomaterials like carbon nanotubes [2,3,4,5,6,7,8], graphene [9,10,11,12,13,14,15], and other 2D structures [16,17,18,19,20,21]. These materials offer remarkable electrical, mechanical, and thermal properties, enabling innovations in transistors, diodes, flexible electronics, etc. As device miniaturization continues, nanoelectronics plays a critical role in the evolution of computing, data storage, and next-generation communication systems, driving advancements in artificial intelligence, the Internet of Things (IoT), and quantum computing. The societal impact of these advances is the rapid transformation of industries through the enabling of faster, more efficient electronic systems, while nanosensors are triggering new progress in healthcare, agriculture, and environmental protection. Indeed, the integration of nanomaterials in sensor technology enables the detection of chemical, biological, and environmental signals with high sensitivity and specificity, opening new opportunities in the field of sensing technology and real-time detection.

In this Special Issue, we bring together contributions that not only push the boundaries of current research but also offer insights into possible future directions for nanotechnology. Our aspiration is for this collection to serves as a valuable resource for researchers, engineers, and industry professionals, inspiring new ideas and fostering collaboration in this field.

In the paper by Hong Yu et al. [22], the authors provide results on the transport properties of wrinkled graphene nanoribbons (WGNR), which could provide guidance for designing flexible graphene-based electronic nanodevices by attaching other similar multidecker metal–arene nanowires. Indeed, they investigated the transport properties of four-terminal vanadium–benzene nanowire V_7_(Bz)_8_-WGNR devices tuned by the polarized gate, and they analyzed the conductance, spin-polarized current, transmission spectra, local density of states, and scattering states. They demonstrate that the tuning effect of V_7_(Bz)_8_ mainly depends on its down-spin state, which can induce a spin-polarized transport property for such devices.

The paper by E.I. Chereches and A.A. Minea [23] focuses on the possible enhancement of the electrical conductivity of polyethylene glycol (PEG 400) nanocolloids through the use of two different kinds of oxide nanoparticle. In particular, two oxides, MgO and TiO_2_, were added to the base fluid in five mass concentrations in the range of 0.25–2.5%wt. The authors have evaluated the stability of oxide–PEG 400 nanocolloids in terms of pH at ambient temperature. Moreover, the electrical conductivity was analyzed at room temperature and when raising the temperature to 333.15 K. It is reported that MgO nanoparticles cause an increase in electrical conductivity larger than thar of TiO_2_ nanoparticles. In particular, 0.25% MgO is responsible for an enhancement of electrical conductivity of 48%. This study opens the way towards future developments in the application of nanocolloids with PEG 400 as the base fluid for practical heat transfer applications at medium temperatures.

In the paper by Sanguk Lee et al. [24], a novel source/drain (S/D) extension scheme to increase the stress in nanosheet (NS) field-effect transistors (NSFETs) is proposed and analyzed using technology computer-aided design simulations. The S/D extension scheme enhances the diffusionless advantage of laser spike annealing. Consequently, higher stress is induced in the NS and carrier mobility is increased. It is reported that the LSA process, when applied to NSFETs, causes a reduction in the on-state current (I_on_). The S/D extension scheme can overcome the current reduction by using an etching process before S/D formation. Considering that a larger S/D volume can induce more stress in the NS channels, in this study, the stress has been boosted by over 25%. Moreover, I_on_ is improved by the increase in carrier concentrations in the NS channels. Finally, an increased I_on_ by 21.7% is reported in NFETs when compared with NSFETs without the proposed scheme. hTe S/D extension scheme solves the I_on_ reduction issue due to LSA and enhances AC/DC performance.

In the paper by Yajun Cai et al. [25], because the heat released in aluminum–air batteries can have a significant impact on the their performance and operating life, the authors carried out a study on the thermal effects of the aluminum–air battery. The authors developed a theoretical model considering different sources of heat during the discharge process, namely, the aluminum oxidation reaction heat at the anode, the cathodic oxygen reduction reaction, the heat production against the battery’s internal resistance, and the hydrogen evolution heat. This study offers valuable insights into the thermal effect analysis of aluminum–air batteries and the management of the thermal process by suppressing hydrogen evolution, thereby enhancing their practical usability. A quantitative analysis of each component revealed that all sources of heat generation rose with increasing discharge current density. Notably, heat production due to hydrogen evolution was the most significant, contributing up to 90%. Moreover, a regulatory strategy to inhibit hydrogen evolution was devised, introducing hybrid additives into the electrolyte and resulting in a reduction in both the hydrogen evolution rate and the associated heat by more than 50%.

In the paper by C. D. K. Mutepfe and V. Srivastava [26], the design and implementation of a graphene-based tunable microwave filter for THz applications is reported. In this study, a reconfigurable substrate-integrated waveguide (SIW) filter designed to operate in the THz region was developed. The filter consisted of two SIW resonators, which were magnetically coupled via an iris, forming a second-order filter structure on a double-layer substrate. The first layer was made of silicon with a permittivity of 11.9, while the second layer was made of silicon dioxide with a permittivity of 3.9. Gold plates were used for both the ground and top planes. Graphene was employed to enable tunability of the filter. A thin graphene sheet was placed between the silicon dioxide layer and the upper gold plate, and an external DC bias voltage was applied to adjust the graphene’s chemical potential. This adjustment allowed the filter’s central operating frequency to shift within the range of 1.289 THz to 1.297 THz, corresponding to a bandwidth of 8 GHz. In the second phase of the research, the aspect ratio of the graphene patch was modified to further tune the center frequency. This approach resulted in frequency changes from 1.2908 THz to 1.2929 THz, yielding a bandwidth shift of 2.1 GHz.

In the paper by E. V. Emelin et al. [27], the formation of diamane nanostructures in bilayer graphene on langasite under irradiation with a focused electron beam is reported. Raman spectra changes and increased electrical resistance after irradiation suggest a local phase transition linked to graphene diamondization. This transition is likely caused by the release of hydrogen and oxygen atoms from PMMA and langasite due to the “knock-on” effect of the electron beam. Theoretical and experimental analyses indicate the formation of sp^3^-hybridized carbon nanoclusters, marking the first instance of local tuneable diamondization in bilayer graphene. Electron beam parameters influenced the phase transition, with a significant increase in resistance post-irradiation. Moreover, the theoretical model closely matched the structural and electronic changes observed in experiments. Finally, transport analysis showed a shift in I–V characteristics from linear to nonlinear after irradiation, indicating a carrier barrier in the affected areas. This was consistent with the phase transition involving hydrogen and oxygen bonding to graphene, forming stable diamane nanoclusters.

In the paper by A. Shoaib et al. [28], a nanotechnology-based approach to biosensor application in current diabetes management practices is reviewed. Early detection and diagnosis are critical for effectively managing various diseases, such as diabetes mellitus. This has led to a notable increase in the utilization of glucose monitoring devices. In particular, biosensors must be user-friendly, highly sensitive, and cost-effective. Recent advancements in biosensor technology have highlighted the potential of nanomaterials in enhancing their performance.

In the paper by Suresh Sagadevan et al. [29], the research activity concerning the sensor and electronics applications of graphene oxide through azobenzene (AZO) grafting is reviewed. The authors summarize recent advancements in graphene-related two-dimensional materials and AZO polymer hybrid structures. AZO polymers are known for their light-induced conformations, rapid response times, photochemical stability, and surface-relief structures, and they represent promising candidates for temperature sensors and light-controllable molecular electronics. While these polymers can endure trans-cis isomerization through light irradiation or heating, they suffer from low photon lifetime and energy density, along with a tendency to agglomerate even at low doping levels, which diminishes their optical sensitivity. Graphene derivatives, such as graphene oxide and reduced graphene oxide, provide an excellent platform for combining with AZO-based polymers to create novel hybrid structures with unique molecular ordering properties. AZO derivatives can enhance energy density, optical responsiveness, and photon storage capacity, potentially mitigating aggregation and reinforcing the AZO complexes. These hybrid materials hold promise for various applications, including sensors, photocatalysts, photodetectors, and photocurrent switching.

## Data Availability

No new data were created or analyzed in this study. Data sharing is not applicable to this article.
